# Stereological Assessment of Locus Coeruleus in the Mouse: A Methodological Study in Pups and Adult Animals

**DOI:** 10.3390/mps9020064

**Published:** 2026-04-09

**Authors:** Marco Scotto, Alessandro Galgani, Marina Boido, Nooria Mohammady, Alessandro Vercelli, Filippo S. Giorgi

**Affiliations:** 1 Sant’Anna School of Advanced Studies, 56125 Pisa, Italy; 2 Department of Translational Research and of New Surgical and Medical Technologies, University of Pisa, 56125 Pisa, Italy; 3 Department of Neuroscience Rita Levi Montalcini, University of Turin, 10126 Turin, Italy; marina.boido@unito.it (M.B.);; 4 Neuroscience Institute Cavalieri Ottolenghi, University of Turin, 10043 Orbassano (TO), Italy; 5 Department of Biology, University of Pisa, 56125 Pisa, Italy; 6 I.R.C.C.S. Fondazione Stella Maris, Calambrone, 56128 Pisa, Italy

**Keywords:** Locus Coeruleus, stereology, mouse brain, noradrenaline, neurodevelopmental disorders, neurodegeneration, infancy, middle age

## Abstract

Unbiased stereology represents the most accurate approach for estimating the total number of neurons of specific brain regions; however, its reliability critically depends on the use of rigorously defined and anatomically appropriate sampling parameters. The brain nucleus Locus Coeruleus (LC) plays a key role in several brain functions. LC impairment has been associated with a range of disorders affecting individuals across the lifespan, from infancy to adulthood. In animal models of these conditions, precise estimation of LC neuronal number is essential. The LC analysis poses specific methodological challenges due to its small size, indistinct anatomical boundaries, and age-dependent changes in neuronal density. In this study, we present a detailed and reproducible stereological workflow for the quantification of LC neurons in the mouse brain across the lifespan. Using C57BL/6J mice at postnatal, adult, and aged stages, we optimized all key components of the Optical Fractionator method, LC neurons were identified by immunoperoxidase staining for tyrosine hydroxylase (TH) and quantified using systematic-random sampling implemented in Stereo Investigator^®^ software. We show that age-specific adjustment of stereological parameters is necessary to obtain reliable estimates, particularly at early postnatal stages characterized by high neuronal packing density. With the optimized protocols described here, TH+ LC neuron counts consistently met accepted precision criteria, as assessed by the Gundersen coefficient of error.

## 1. Introduction

The Locus Coeruleus (LC), the main noradrenergic (NA) nucleus of the central nervous system (CNS), is a key modulator of neuronal networks and plays a central role in maintaining brain homeostasis [1]. The LC is a major hub of the sleep/wake cycle circuitry, promotes attentional focusing and shifting, and modulates autonomic brain centers [2,3,4]. At the same time, NA released by LC axons acts not only as a neurotransmitter but also as a paracrine hormone, modulating microglial activity, regulating cerebral blood flow, and promoting the production of neuronal growth factors [5,6,7,8].

Despite its crucial roles, the LC appears particularly vulnerable to damage and functional impairment and is implicated in a wide range of neurological disorders affecting different age groups [9,10,11,12]. For instance, LC dysregulation and hyperactivity have been reported in autism spectrum disorders and attention-deficit hyperactivity disorder [9,11]; moreover, LC lesions have been observed in cases of sudden infant death and perinatal hypoxic injury [13,14]. In neurodegenerative disorders such as Alzheimer’s Disease and Parkinson’s Disease, the LC undergoes early and severe degeneration, likely contributing to disease pathogenesis by exacerbating neuroinflammatory activation, altering neurovascular function, and promoting the spreading and accumulation of neurotoxic proteins [10,12,15].

For these reasons, LC has been the focus of numerous studies, both in clinical settings—largely relying on neuroimaging approaches—and in mouse models [10,16,17,18,19,20,21]. A major challenge of these studies has been the accurate estimation of LC morphological and/or functional integrity. In clinical investigations, direct measurements are virtually impossible, requiring indirect post-acquisition analyses of radiological images [22,23], whereas in animal models, brain tissue can be directly collected and examined. To date, the most accurate and refined method for estimating LC integrity is unbiased stereological analysis, which provides an estimate of the total number of NA neurons within the anatomical nucleus [24,25,26,27,28].

Although unbiased stereology is a reliable approach, it requires a precisely defined and reproducible workflow that accounts for both anatomical and methodological variables [25,26,27,28]. These include LC-specific features such as its boundaries and the density and spatial distribution of NA neurons, as well as technical aspects of tissue processing and analysis. Importantly, LC anatomy varies markedly across experimental species and, within the same species, across different ages [29,30]. To accommodate this variability, stereological protocols must be specifically adapted in order to ensure accurate and comparable results.

In this paper, we present the stereological protocols we developed for LC analysis in mice at different ages, describing key methodological and analytical details.

## 2. Experimental Design

Experimental procedures and methodological optimization were established in the C57BL/6J mouse strain, one of the most widely used models in neuroscientific research [31]. To account for age-related variability, animals were analyzed at postnatal day 12 (PN12; pups), 4 months (young adults), and 10 months of age (middle-aged). These time points allow the analysis of LC development from early postnatal stages through late adulthood. Mice were housed under controlled conditions (22–24 °C) with a 12/12 h light/dark cycle and ad libitum access to food and water. PN12 mice were housed with their dams until sacrifice. No animals were excluded from the experimental procedure. All experimental procedures were performed in compliance with national (D.L. N.26, 4 March 2014) and international Directives and Laws (Directive 2010/63/EU). The animal study protocol was approved by the University of Pisa OPBA and Italian Ministry of Health (authorization # 961/2024-PR; 8 October 2024).

LC NA neurons were identified by immunoperoxidase staining for tyrosine hydroxylase (TH), the rate-limiting enzyme in noradrenaline synthesis and a well-established marker for LC neuron identification [32,33].

Unbiased stereological cell counting was performed using a Nikon Eclipse Ni microscope (Nikon, Tokyo, Japan) equipped with a motorized stage (Prior Scientific, Fulbourn, UK) and Stereo Investigator^®^ software version 2024.1.3 (MBF Bioscience, Delft, The Netherlands).

### 2.1. Materials

1. Paraformaldehyde 95% (Sigma-Aldrich, Darmstadt, Germany; Cat. no.: 158127)
2. Alcoolpath 100 (Bio-Optica Milano S.p.A, Milan, Italy; Cat. no.: 06-10030F)
3. Xylene (Bio-Optica Milano S.p.A, Milan, Italy; Cat. no.: 06-1304F)
4. Biowax (Bio-Optica Milano S.p.A, Milan, Italy; Cat. no.: 08-7960)
5. Feather Blades A22 (Bio-Optica Milano S.p.A, Milan, Italy; Cat. no.: 01-A22)
6. Adhesive Plus slides (Bio-Optica Milano S.p.A, Milan, Italy; Cat. no.: 09-3000)
7. A-PapPen (Bio-Optica Milano S.p.A, Milan, Italy; Cat. no.: 11-100)
8. Triton X-100 (Sigma-Aldrich, Darmstadt, Germany; Cat. no.: X-100)
9. Trizma (Sigma-Aldrich, Darmstadt, Germany; Cat. no.: T1503)
10. Normal Goat Serum (Vector Laboratories, Inc., Newark, CA, USA; Cat. no.: S-1000)
11. Primary antibody, anti-tyrosine hydroxylase (Sigma-Aldrich, Darmstadt, Germany; Cat. no.: Ab152)
12. Goat anti-Rabbit biotinylated secondary antibody (Vector Laboratories, Inc., Newark, CA, USA; Cat. no.: BA-1000)
13. Vectastain^®^ Elite^®^ ABC Universal PLUS Kit Peroxidase (Vector Laboratories, Inc., Newark, CA, USA; Cat. no.: PK-8200)
14. DAB Substrate Kit Peroxidase (HRP) (Vector Laboratories, Inc., Newark, CA, USA; Cat. no.: SK-4100)
15. DPX Histology Mounting Medium (Sigma-Aldrich, Darmstadt, Germany; Cat. no.: 06522)

### 2.2. Equipment

1. Thermal Unit UT200 (Bio-Optica Milano S.p.A, Milan, Italy)
2. Paraffin Dispenser DP500 (Bio-Optica Milano S.p.A, Milan, Italy)
3. Rotary microtome (HistoCore BIOCUT, Leica Biosystems, Deer Park, IL, USA)
4. Forced-air oven (Falc Instruments s.r.l., Treviglio (BG), Italy)
5. Nikon Eclipse Ni microscope (Nikon, Japan)
6. Motorized stage (Prior Scientific, UK)
7. Stereo Investigator^®^ software (MBF Bioscience, The Netherlands)

## 3. Procedure

To provide a detailed description of the protocol developed and validated for counting TH+ neurons of the LC, we divided this section into three main parts, with the second one being focused on the staining procedure, while the latter is dedicated to stereological assessment, which represents the main focus of the study.

### 3.1. Embedding and Cutting Procedure

1. After sacrifice, fix brains in 4% Paraformaldehyde in phosphate buffer (pH 7.0) overnight at 4 °C.
2. Wash samples in phosphate buffer two times (pH 7.0).
3. Dehydrate samples through a graded ethanol series (30% and 50%, 1.5 h for each step), followed by 80% ethanol step overnight at 4 °C.
4. Complete dehydration with a 2 h step in 90% ethanol, followed by a 3 h step in absolute ethanol.
5. After dehydration procedure, immerse brains in xylene (two changes, 2.5 h each) to allow paraffin infiltration.
6. Once clearing in xylene is complete, transfer the samples to melted paraffin (58–60°) overnight in Thermal Unit UT200 (Bio-Optica Milano S.p.A, Milan, Italy).
7. Embed the brains in paraffin using metal molds, profiting from Paraffin Dispenser DP500 (Bio-Optica Milano S.p.A, Milan, Italy).
8. Section each paraffin-embedded brain along the rostro-caudal axis at the level of the brainstem to obtain 40 µm thick coronal sections using a rotary microtome (HistoCore BIOCUT, Leica Biosystems, USA).
9. Collect sections onto adhesive plus slides and dry them overnight in an oven at 37 °C.

### 3.2. Immunoperoxidase for TH+

1. Identify coronal sections containing LC from Bregma −5.80 mm, Interaural −2.00 mm to Bregma −5.34 mm, Interaural −1.54 mm, according to the Paxinos & Franklin mouse brain atlas [34].
2. Deparaffinize the selected slides in xylene and subsequently rehydrate through a graded series of ethanol to distilled water.
3. Permeabilize coronal sections using 0.1% Triton X-100 in Tris Buffer Saline (pH 7.0) for 15 min.
4. Following permeabilization, quench endogenous peroxidase activity by incubating sections in a 3% aqueous solution of hydrogen peroxide for 10 min.
5. Block non-specific binding sites using 10% Normal Goat Serum (NGS) in TBS 1X for 1 h at Room Temperature.
6. Incubate sections with a rabbit anti-tyrosine hydroxylase primary antibody (1:1000, Sigma-Aldrich, Darmstadt, Germany; Cat. no.: Ab152) in TBS 1X containing 2% NGS at 4 °C.
7. Following overnight incubation, rinse sections in TBS three times and then incubate with a biotinylated goat anti-Rabbit IgG (H + L) secondary antibody (1:400, Vector Laboratories, Inc., Newark, CA, USA; Cat. no.: BA-1000) for 1.30 h at room temperature.
8. Rinse sections in TBS 1X three times. Incubate samples with avidin–biotin complex solution using Vectastain^®^ Elite^®^ ABC Universal PLUS Kit Peroxidase (Vector Laboratories, Inc., Newark, CA, USA; Cat. no.: PK-8200) according to the manufacturer’s instructions for 1 h at room temperature.
9. Rinse sections in TBS 1X three times. Incubate with DAB Substrate Kit Peroxidase (HRP) (Vector Laboratories, Inc., Newark, CA, USA; Cat. no.: SK-4100). The enzymatic reaction is catalyzed by horseradish peroxidase (HRP) using 3,3′-Diaminobenzidine as the chromogenic substrate.

### 3.3. Stereological Assessment

**Figure 1:**
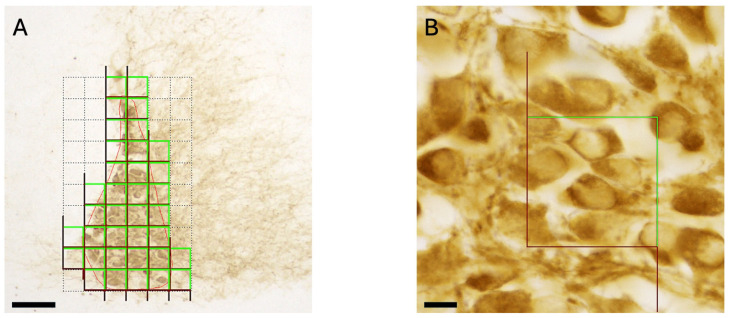
Representative image of area sampling fraction (ASF) applied to the drawn contour of a section from a PN12 mouse brain, ((**A**) scale bar 80 μm). Representative image of a counting frame (CF) during randomized sampling, ((**B**) scale bar 10 µm).

Stereological analysis was performed using the Optical Fractionator (OF) method implemented in Stereo Investigator^®^ (MBF Bioscience, The Netherlands). The accuracy and reliability of this approach critically depend on the sampling scheme, which must be carefully optimized according to the specific anatomical and cytoarchitectural features of the target region. A central challenge in protocol development is achieving an optimal balance between measurement precision and sampling effort, with the goal of maximizing accuracy while minimizing the number of sections and counting sites required. To achieve this aim, OF requires the operator to follow these steps:

- **Contour delineation:** At low magnification, the region of interest (ROI) is outlined to define the anatomical boundaries of the target structure (here, the LC). Accurate contour delineation is essential, as the ROI defines the reference space for all subsequent sampling steps. Ambiguous or inconsistently defined borders may introduce systematic bias and adversely affect the validity of the stereological estimates.
- **Counting frame (CF):** The CF represents the two-dimensional sampling probe used for cell counting within the ROI. It is defined in the x–y plane and applied following unbiased counting rules. In particular, the CF is defined by two green borders and two red borders (Figure 1A,B). Only objects (here, TH+ neurons) located within the CF that do not touch any border or cross the green lines are counted, whereas those touching the red lines are excluded. This approach is mandatory to avoid counting bias, especially double counting. CF dimensions are adjusted so that, on average, each frame contains approximately 3–6 countable neurons, ensuring efficient sampling while maintaining acceptable variance (Figure 1B).
- **Area sampling fraction (ASF):** The ASF defines the proportion of the ROI area that is sampled and is determined by the ratio between the counting frame area and the x–y step length. ASF is selected based on the spatial distribution of neurons within the ROI. Relatively homogeneous distributions allow for lower ASF values, whereas heterogeneous or clustered distributions require higher ASF to compensate for increased variance. Counting frames are positioned in a systematic-random manner across the ROI by the software (Figure 1A).
- **Optical dissector height and guard zones:** three-dimensional sampling is achieved using the optical dissector probe along the z-axis. The dissector height is defined within the measured section thickness and is chosen based on both tissue integrity and the axial distribution of neurons. Guard zones are applied at the upper and lower surfaces of the section to avoid counting artifacts caused by sectioning-induced cell loss or compression. The guard zone width should be determined based on a number of parameters, including section thickness, the consistency of thickness across slides, and the size of the counting unit.
- **Section sampling fraction (SSF):** the section sampling fraction determines the proportion of serial sections analyzed throughout the rostro-caudal extent of the ROI. Based on the size and anatomical continuity of the target structure (here, the LC), a systematic-random series of sections is selected for analysis. The SSF is expressed as an interval (e.g., 1:3), indicating that one section is analyzed every three serial sections, with the starting section randomly selected. Cell numbers in non-sampled sections are estimated from sampled sections. As with ASF, SSF is adjusted according to the degree of regularity or heterogeneity in neuronal distribution along the anatomical axis.

Once all stereological parameters are defined, cell counting can be performed. The accuracy and reliability of the estimates are evaluated by comparison with values reported in the literature and by assessing the Gundersen coefficient of error (CE) [35,36,37]. The Gundersen CE provides an estimate of the precision of a stereological measurement by quantifying the variance introduced by the sampling scheme, independent of biological variability. In general, stereological estimates are considered reliable when the Gundersen CE is below 0.10 [35].

#### Stereology Protocol Implementation and Reliability Assessment

Based on previous studies [24], we defined the stereology protocol for four-month-old mice (N = 3) as follows:

- SSF: 1:3
- SAF: 90% of sampling area
- CF: 60 μm × 60 μm
- Dissector height and top guard zone: 12 μm + 3μm.

Fully stained, clearly identifiable TH+ neuronal somata with a size > 10 µm were considered as counting units. TH+ neurons were counted at 100× OIL magnification, and the thickness of each section was remeasured to improve the accuracy of stereological estimation.

Using this protocol, we obtained a mean TH+ count of 3064 ± 729.6, with a Gundersen CE consistently < 0.1. The same reliability was found in the ten-month-old mice (N = 3), with a mean TH+ count of 3328 ± 218.8 and a Gundersen CE consistently < 0.1.

When we applied the same protocol to PN12 pups (N = 3), estimations were not reliable, as Gundersen CE were consistently > 0.1. Thus, we modified the parameters as follows:

- SSF: 1:2
- SAF: 90% of sampling area
- CF: 40 μm × 40 μm
- Dissector height and top guard zone: 15 μm + 3μm.

**Figure 2:**
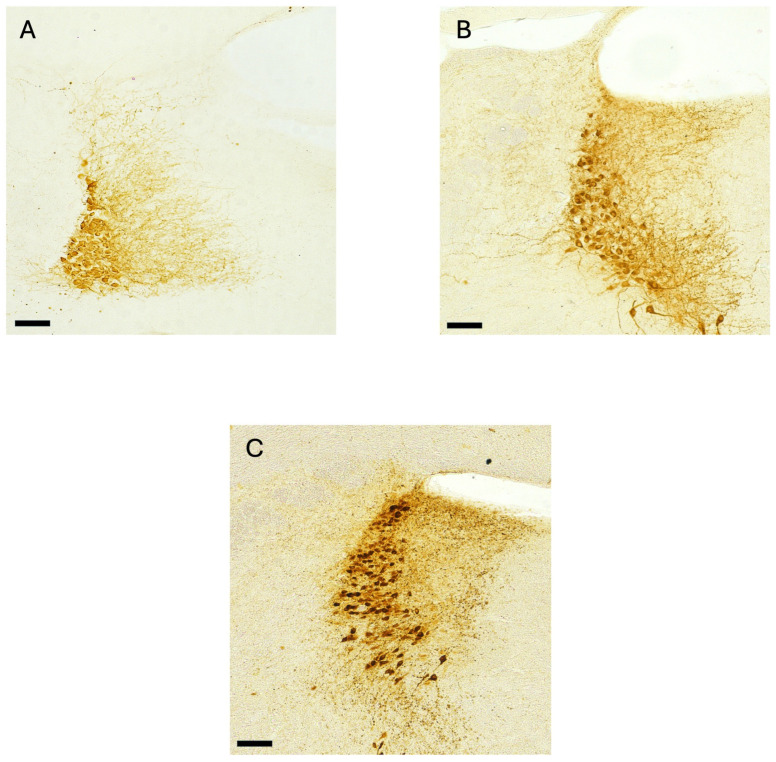
Representative images of the Locus Coeruleus region from approximately the midsection along the rostro-caudal axis of the pons in PN12 pups (**A**), four-month-old (**B**), and ten-month-old (**C**) mice. The reported scale bar is 50 µm.

The modifications were performed in light of the morphological characteristics of the LC neurons at this postnatal age, where neurons are more densely packed compared with those observed in adult animals (Figure 2). Therefore, the counting frame size was reduced to include a maximum of 6 neurons. Additionally, dissector height was increased from 12 μm to 15 μm to ensure sufficient sampling volume while maintaining accurate discrimination between individual neurons.

Using this protocol, we obtained a mean TH+ count of 2272 ± 79.97, with a Gundersen CE consistently < 0.1.

## 4. Discussion

In this methodological study, we describe the stereological protocols we developed for the quantitative assessment of TH+ neurons within the LC of pups and adult mice. The procedures yielded highly consistent reliability across samples, demonstrating robustness and reproducibility when systematically applied.

Among the available stereological approaches, we selected the Optical Fractionator (OF) with systematic-random sampling as implemented in MBF^®^ Stereo Investigator [27]. The OF, developed in the 1980s within the framework of design-based stereology, enables unbiased estimation of total neuronal number independent of assumptions regarding cell size, shape, or orientation [25,36]. In contrast to earlier correction-factor approaches [38,39], the OF does not rely on geometric characteristics of the specimen to mathematically compensate for sampling bias [36,37]. Instead, total number is derived exclusively from known sampling fractions in the *x*–*y* and *z* dimensions [36,37].

Classical or model-based stereological methods—including the Abercrombie correction and subsequent refinements—estimate total number by applying mathematical correction factors to 2D profile counts [38,39]. These corrections require measurements of structural parameters such as section thickness, particle height (e.g., soma or nuclear diameter), and the minimal portion of the particle required to penetrate the section (“lost caps”) [38]. Even in their more sophisticated formulations (e.g., size-weighted corrections), these approaches remain dependent on assumptions about particle geometry and distribution [39]. Inclusion probability is therefore indirectly inferred from morphological measurements and may vary systematically when particle size is heterogeneous or altered by experimental conditions [40]. As a consequence, any biological factor affecting soma size or shape can influence the correction factor itself, potentially introducing group-dependent bias [38,39,40].

Conversely, design-based stereology, on which the OF is based, overcomes these limitations by defining a three-dimensional sampling probe *a priori* [25,27,36,37]. The introduction of an optical dissector height, guard zones, and strict inclusion/exclusion rules ensures that each neuron has a known and equal probability of being sampled [28]. Importantly, inclusion probability depends only on the sampling design (section sampling fraction, area sampling fraction, and thickness sampling fraction), not on the morphological characteristics of the neurons [28]. The systematic-random sampling scheme further guarantees uniform coverage of the entire structure while minimizing variance; in this framework, bias is prevented structurally rather than corrected mathematically [27].

These methodological considerations are particularly relevant for LC-NA neurons. LC neurons exhibit variability in soma size and are unevenly distributed along the rostro-caudal axis as well as within individual sections [8,41,42]. Moreover, neuronal morphology may be altered in developmental stages or under pathological conditions, including chemical lesions or neurodegenerative processes [19,43]. In such contexts, size-dependent correction methods become especially vulnerable, because the correction factor itself may change as a function of disease-related morphological alterations. For studies aimed at detecting subtle changes in LC integrity, reliance on model-based corrections could therefore confound biological effects with geometric assumptions.

By contrast, the OF provides a size-independent estimate of total TH+ neuron number, making it particularly suitable for comparative studies across age groups and experimental models. Because the method directly samples in three dimensions and does not extrapolate from 2D intersections, it avoids the need for assumptions regarding uniform size, isotropy, or distribution.

Finally, it may be worth briefly commenting on the results obtained in the groups assessed using our counting protocols. We observed a lower LC cell count in pups, whereas adult mice at both 4 and 10 months of age showed higher mean estimates. These data may reflect the age-related increase in TH+ neurons in the LC observed during postnatal development, consistent with the continued differentiation and maturation of the LC after birth in rodents [9]. However, given the limited sample size and the high variability of the LC population, we cannot support this inference with robust statistical analysis, nor can we meaningfully interpret the slight difference observed between 4- and 10-month-old mice. Accordingly, further interpretation of these findings is beyond the scope of this primarily methodological study.

In conclusion, we developed and validated two design-based stereological protocols tailored to LC TH+ neuron quantification in pups and adult mice. These protocols provide reliable, assumption-minimized estimates of neuronal number and are well-suited for future investigations of LC alterations under physiological and pathological conditions. Given the structural variability of the LC and its susceptibility to developmental and degenerative changes, we consider unbiased, design-based stereology to represent the most appropriate methodological framework for quantitative LC assessment in the mouse model.

## Data Availability

The data supporting the findings of this study are available from the corresponding author upon reasonable request.

## Abbreviations

The following abbreviations are used in this manuscript:

ASF	Area Sampling Fraction
CE	Coefficient of Error
CF	Counting Frame
CNS	Central Nervous System
DAB	3,3′-Diaminobenzidine
HRP	Horseradish Peroxidase
LC	Locus Coeruleus
NA	Noradrenaline
NGS	Normal Goat Serum
OF	Optical Fractionator
PN12	Postnatal Day 12
ROI	Region of Interest
SSF	Section Sampling Fraction
TBS	Tris Buffer Saline
TH	Tyrosine Hydroxylase

## References

[1] Poe G.R., Foote S., Eschenko O., Johansen J.P., Bouret S., Aston-Jones G., Harley C.W., Manahan-Vaughan D., Weinshenker D., Valentino R. (2020). Locus Coeruleus: A New Look at the Blue Spot. Nat. Rev. Neurosci..

[2] Giorgi F.S., Galgani A., Puglisi-Allegra S., Busceti C.L., Fornai F. (2021). The Connections of Locus Coeruleus with Hypothalamus: Potential Involvement in Alzheimer’s Disease. J. Neural Transm..

[3] Aston-Jones G., Waterhouse B. (2016). Locus Coeruleus: From Global Projection System to Adaptive Regulation of Behavior. Brain Res..

[4] González M.M.C., Aston-Jones G. (2006). Circadian Regulation of Arousal: Role of the Noradrenergic Locus Coeruleus System and Light Exposure. Sleep.

[5] Giorgi F.S., Galgani A., Puglisi-Allegra S., Limanaqi F., Busceti C.L., Fornai F. (2020). Locus Coeruleus and Neurovascular Unit: From Its Role in Physiology to Its Potential Role in Alzheimer’s Disease Pathogenesis. J. Neurosci. Res..

[6] Giorgi F.S., Saccaro L.F., Galgani A., Busceti C.L., Biagioni F., Frati A., Fornai F. (2019). The Role of Locus Coeruleus in Neuroinflammation Occurring in Alzheimer’s Disease. Brain Res. Bull..

[7] Feinstein D.L., Kalinin S., Braun D. (2016). Causes, Consequences, and Cures for Neuroinflammation Mediated via the Locus Coeruleus: Noradrenergic Signaling System. J. Neurochem..

[8] Schwarz L.A., Luo L. (2015). Organization of the Locus Coeruleus-Norepinephrine System. Curr. Biol..

[9] Galgani A., Bartolini E., D’Amora M., Faraguna U., Giorgi F.S. (2023). The Central Noradrenergic System in Neurodevelopmental Disorders: Merging Experimental and Clinical Evidence. Int. J. Mol. Sci..

[10] Galgani A., Giorgi F.S. (2023). Exploring the Role of Locus Coeruleus in Alzheimer’s Disease: A Comprehensive Update on MRI Studies and Implications. Curr. Neurol. Neurosci. Rep..

[11] Bast N., Poustka L., Freitag C.M. (2018). The Locus Coeruleus–Norepinephrine System as Pacemaker of Attention–a Developmental Mechanism of Derailed Attentional Function in Autism Spectrum Disorder. Eur. J. Neurosci..

[12] Beardmore R., Hou R., Darekar A., Holmes C., Boche D. (2021). The Locus Coeruleus in Aging and Alzheimer’s Disease: A Postmortem and Brain Imaging Review. J. Alzheimer’s Dis..

[13] Lavezzi A.M., Alfonsi G., Matturri L. (2013). Pathophysiology of the Human Locus Coeruleus Complex in Fetal/Neonatal Sudden Unexplained Death. Neurol. Res..

[14] Pagida M.A., Konstantinidou A.E., Korelidou A., Katsika D., Tsekoura E., Patsouris E., Panayotacopoulou M.T. (2016). The Effect of Perinatal Hypoxic/Ischemic Injury on Tyrosine Hydroxylase Expression in the Locus Coeruleus of the Human Neonate. Dev. Neurosci..

[15] Oertel W.H., Henrich M.T., Janzen A., Geibl F.F. (2019). The Locus Coeruleus: Another Vulnerability Target in Parkinson’s Disease. Mov. Disord..

[16] Jacobs H.I.L., Becker J.A., Kwong K., Engels-Domínguez N., Prokopiou P.C., Papp K.V., Properzi M., Hampton O.L., d’Oleire Uquillas F., Sanchez J.S. (2021). In Vivo and Neuropathology Data Support Locus Coeruleus Integrity as Indicator of Alzheimer’s Disease Pathology and Cognitive Decline. Sci. Transl. Med..

[17] Galgani A., Lombardo F., Martini N., Vergallo A., Bastiani L., Hampel H., Hlavata H., Baldacci F., Tognoni G., De Marchi D. (2023). Magnetic Resonance Imaging Locus Coeruleus Abnormality in Amnestic Mild Cognitive Impairment Is Associated with Future Progression to Dementia. Eur. J. Neurol..

[18] Aghakhanyan G., Galgani A., Vergallo A., Lombardo F., Martini N., Baldacci F., Tognoni G., Leo A., Guidoccio F., Siciliano G. (2023). Brain Metabolic Correlates of Locus Coeruleus Degeneration in Alzheimer’s Disease: A Multimodal Neuroimaging Study. Neurobiol. Aging.

[19] Heneka M.T., Ramanathan M., Jacobs A.H., Dumitrescu-Ozimek L., Bilkei-Gorzo A., Debeir T., Sastre M., Galldiks N., Zimmer A., Hoehn M. (2006). Locus Ceruleus Degeneration Promotes Alzheimer Pathogenesis in Amyloid Precursor Protein 23 Transgenic Mice. J. Neurosci..

[20] Jardanhazi-Kurutz D., Kummer M.P., Terwel D., Vogel K., Dyrks T., Thiele A., Heneka M.T. (2010). Induced LC Degeneration in APP/PS1 Transgenic Mice Accelerates Early Cerebral Amyloidosis and Cognitive Deficits. Neurochem. Int..

[21] Heneka M.T., Nadrigny F., Regen T., Martinez-Hernandez A., Dumitrescu-Ozimek L., Terwel D., Jardanhazi-Kurutz D., Walter J., Kirchhoff F., Hanisch U.K. (2010). Locus Ceruleus Controls Alzheimer’s Disease Pathology by Modulating Microglial Functions through Norepinephrine. Proc. Natl. Acad. Sci. USA.

[22] Trujillo P., Petersen K.J., Cronin M.J., Lin Y.C., Kang H., Donahue M.J., Smith S.A., Claassen D.O. (2019). Quantitative Magnetization Transfer Imaging of the Human Locus Coeruleus. Neuroimage.

[23] Trujillo P., Aumann M.A., Claassen D.O. (2024). Neuromelanin-Sensitive MRI as a Promising Biomarker of Catecholamine Function. Brain.

[24] Zhu Y., Fenik P., Zhan G., Somach R., Xin R., Veasey S. (2016). Intermittent Short Sleep Results in Lasting Sleep Wake Disturbances and Degeneration of Locus Coeruleus and Orexinergic Neurons. Sleep.

[25] West M.J., Gundersen H.J.G. (1990). Unbiased Stereological Estimation of the Number of Neurons in the Human Hippocampus. J. Comp. Neurol..

[26] Abusaad I., Mackay D., Zhao J., Stanford P., Collier D.A., Everall I.P. (1999). Stereological Estimation of the Total Number of Neurons in the Murine Hippocampus Using the Optical Disector. J. Comp. Neurol..

[27] West M.J. (2012). Introduction to Stereology. Cold Spring Harb. Protoc..

[28] West M.J., Slomianka L., Gundersen H.J.G. (1991). Unbiased Stereological Estimation of the Total Number of Neurons in the Subdivisions of the Rat Hippocampus Using the Optical Fractionator. Anat. Rec..

[29] Counts S.E., Mufson E.J., Mai J.K., Paxinos G. (2012). Locus Coeruleus. The Human Nervous System.

[30] Galgani A., Scotto M., Faraguna U., Giorgi F.S. (2025). Fading Blue: Exploring the Causes of Locus Coeruleus Damage Across the Lifespan. Antioxidants.

[31] Zhong M.Z., Peng T., Duarte M.L., Wang M., Cai D. (2024). Updates on Mouse Models of Alzheimer’s Disease. Mol. Neurodegener..

[32] Kaufman S. (2006). Tyrosine Hydroxylase. Adv. Enzymol. Relat. Areas Mol. Biol..

[33] Bezin L., Marcel D., Debure L.I., Ginovart N., Rousset C., Pujol J.F., Weissmann D. (1994). Postnatal Development of the Tyrosine Hydroxylase-Containing Cell Population within the Rat Locus Coeruleus: Topological Organization Andphenotypic Plasticity. J. Neurosci..

[34] Paxinos G., Franklin K.B.J. (2019). Paxinos and Franklin’s The Mouse Brain in Stereotaxic Coordinates.

[35] Gundersen H.J.G., Jensen E.B.V., Kiêu K., Nielsen J. (1999). The Efficiency of Systematic Sampling in Stereology-Reconsidered. J. Microsc..

[36] Gundersen H.J.G., Jensen E.B. (1987). The Efficiency of Systematic Sampling in Stereology and Its Prediction. J. Microsc..

[37] Glaser E.M., Wilson P.D. (1998). The Coefficient of Error of Optical Fractionator Population Size Estimates: A Computer Simulation Comparing Three Estimators. J. Microsc..

[38] Abercrombie M. (1946). Estimation of Nuclear Population from Microtome Sections. Anat. Rec..

[39] Clarke P.G.H. (1993). An Unbiased Correction Factor for Cell Counts in Histological Sections. J. Neurosci. Methods.

[40] Hedreen J.C. (1998). What Was Wrong With the Abercrombie and Empirical Cell Counting Methods? A Review. Anat. Rec..

[41] Vreven A., Aston-Jones G., Pickering A.E., Poe G.R., Waterhouse B., Totah N.K. (2024). In Search of the Locus Coeruleus: Guidelines for Identifying Anatomical Boundaries and Electrophysiological Properties of the Blue Spot in Mice, Fish, Finches, and Beyond. J. Neurophysiol..

[42] McKinney A., Hu M., Hoskins A., Mohammadyar A., Naeem N., Jing J., Patel S.S., Sheth B.R., Jiang X. (2023). Cellular Composition and Circuit Organization of the Locus Coeruleus of Adult Mice. eLife.

[43] Kelberman M.A., Rorabaugh J.M., Anderson C.R., Marriott A., DePuy S.D., Rasmussen K., McCann K.E., Weiss J.M., Weinshenker D. (2023). Age-Dependent Dysregulation of Locus Coeruleus Firing in a Transgenic Rat Model of Alzheimer’s Disease. Neurobiol. Aging.

